# Validation and Evaluation of Lateral Flow Tests for the Detection of Antimicrobial Residues on Poultry Feathers

**DOI:** 10.1111/jvp.70000

**Published:** 2025-06-03

**Authors:** Sophie Hedges, Sophie Mompelat, Dominique Hurtaud‐Pessel, Damer P. Blake, Guillaume Fournié, Ludovic Pelligand

**Affiliations:** ^1^ The Royal Veterinary College Hatfield UK; ^2^ London School of Hygiene & Tropical Medicine London UK; ^3^ ANSES Laboratory of Fougeres Javené France; ^4^ INRAE Javené France

**Keywords:** antibiotics, chicken, LFT, liquid chromatography tandem mass spectrometry (LC–MS/MS), veterinary drugs

## Abstract

Persistence of antimicrobial drugs (AMDs) administered to poultry is longer in feathers than in edible tissues. Hence, poultry feathers are a suitable matrix to investigate historical exposure contributing to antimicrobial resistance, since current detection methods are either non‐specific or highly technical and costly. Here we present an analysis of the performance of lateral flow test (LFT) panels in the detection of five AMD classes, namely sulfonamides, tetracyclines, beta‐lactams, quinolones, and aminoglycosides, on chicken feather samples. The limit of detection (LOD) of eight AMD substances was determined between 4.7 μg/kg for enrofloxacin and 700 μg/kg for streptomycin. The performance of feather LFT was evaluated for four AMD classes against the reference method (LC–MS/MS). From 79 samples collected from the field, LFT test specificity ranged from 0.63 (quinolones) to 0.95 (tetracyclines). Test sensitivity ranged from 0.15 (beta‐lactams) to 0.78 (quinolones and tetracyclines). LFT testing had the greatest discriminatory power for tetracyclines (specificity 0.95 and sensitivity 0.78). LFT had similar test characteristics for sulfonamides and quinolones and performed poorly for beta‐lactams. Poor recovery rates (< 15%) were observed in neomycin, kanamycin, and ampicillin. These methods are suitable for preliminarily screening tetracyclines, sulfonamides, and quinolones, with recommendations for further extraction protocols.

## 1 Introduction

Antimicrobial residues are commonly monitored in edible tissues such as meat to ensure adherence to maximum residue limits (MRLs) (Donoghue 2003) and protect consumer health. However, these residues typically deplete quickly after treatment ends and provide limited insight into AMD exposure during the production cycle. In contrast, feathers have been shown to retain residues of certain AMD classes for longer durations, offering potential as a non‐invasive matrix to reconstruct treatment histories. These approaches offer an opportunity to gain a more comprehensive understanding of the potential impact of AMD exposure on AMR emergence patterns.

AMDs used in the poultry industry to prevent and treat diseases accumulate in feathers; therefore, feathers can provide historical information on AMD exposure throughout the production cycle (Berendsen et al. 2013; Jansen et al. 2017; Dreano et al. 2021). Moreover, containing high levels of proteins and amino acids, (Peng et al. 2019) feathers are often ground and used as a by‐product (feather meal) in animal feed and fertilizers (Karuppannan et al. 2021). This practice could increase the amount of AMD residues entering other food production systems (e.g., pig or fish) and the environment.

Lateral flow tests (LFTs) or immuno‐chromatographic tests are fast and simple tools used in diagnostics to detect the presence or absence of a specific target in a sample. In recent years, there has been a notable adaptation of LFTs to specifically target the detection of AMDs in milk samples (Douglas et al. 2012; Naik et al. 2017), with a primary focus on ensuring food safety.

Here, we evaluate the use of the Charm (Charm Sciences, Lawrence, MA, USA) ROSA (Rapid One Step Assay) QUAD1 and QUAD3 lateral flow strips for their use as detection methods of AMDs on poultry feathers.

The objectives of this study were to firstly determine the limit of detection (LOD) of AMDs in spiked buffer and spiked feather samples using analytical standards of drugs commonly used during poultry production. Secondly, we determined the short‐term stability of AMDs on feather samples when reproducing shipment conditions (temperature and time). Finally, we evaluated test sensitivity and test specificity of the LFTs against targeted high‐performance liquid chromatography coupled to tandem mass spectrometry (Dreano et al. 2021) for sulfonamides, tetracyclines, and quinolones, using 79 field samples.

## 2 Materials and Methods

### 2.1 Reagents and Chemical Standards

**FIGURE 1:**
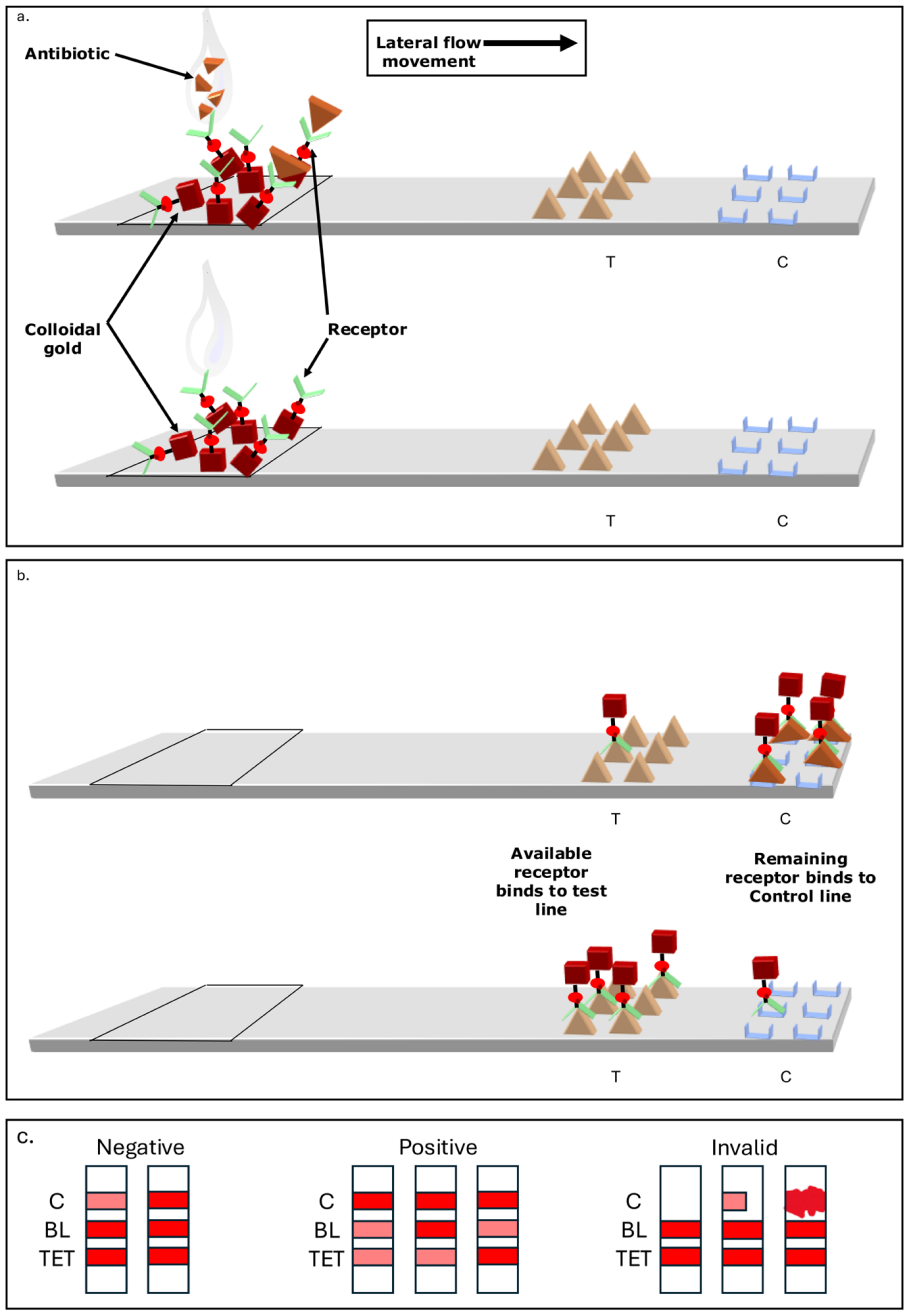
Schematic illustration of the Charm ROSA (Rapid One‐Step Assay) lateral flow test strip used for the detection of AMDs. The top lateral flow test strip in the first two panels (a, b) shows where an AMD is present in the sample and the bottom strip where no AMDs are present. The final panel shows what would be seen on the lateral flow test strip by eye for a negative (left), positive (middle), and invalid sample (right) run respectively. For the negative strips, both beta‐lactams (BL) and tetracyclines (TET) are negative in both instances. For the three positive results, the first strip is positive for BL and TET, the second positive for TET, and the third positive for BL. The final invalid strips are due to the poor formation of the control line. BL, beta‐lactam; *C*, control line; *T*, test line; TET, tetracycline. Adapted from Charm ROSA Principle (Charm Sciences, Lawrence, MA, USA).

Two commercial Rapid One Step Assay (ROSA) lateral flow tests were evaluated (Charm Sciences, Lawrence, MA, USA). QUAD1 (panel 1) is designed for the detection of tetracyclines, quinolones, beta‐lactams, and sulfonamides, and QUAD3 (panel 2) for the detection of aminoglycosides, specifically spectinomycin, neomycin, kanamycin, and streptomycin. They are competitive LFTs, where sample antibiotics (antigen) compete with a reference (test line) for binding to a limited amount of labelled antibody (Figure 1). The cross‐reactivity of the LFT antibodies to various AMDs and related compounds has been assessed by Charm Sciences, ensuring the specificity of the test for the intended analytes. Analytical standards (purity %) of enrofloxacin (99.3%), oxytetracycline (96.3%), ampicillin (91.5%), sulfadiazine (99.56%), streptomycin (96.7%), neomycin (88.2%), kanamycin (76.0%), and spectinomycin (97.2%) were purchased from GLS (Glentham Lab Sciences, Corsham, UK). Individual standard solutions were prepared and stored according to stability data determined by Gaugain et al. (2013) (Appendix S1).

### 2.2 Protocol for Feather Analysis Using Rapid One Step Assay (ROSA) Lateral Flow Test Strips

**FIGURE 2:**
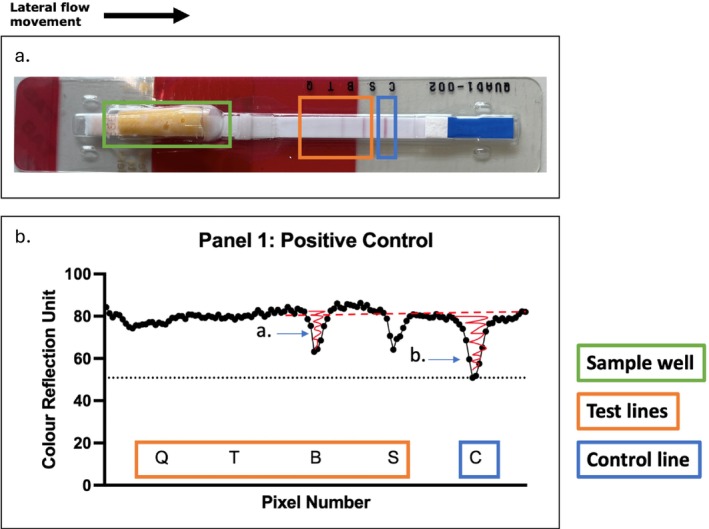
(a) A photograph of a positive control lateral flow test strip for panel 1. (b) Colour Reflection Unit (*Y*‐axis) along pixel number (*X*‐axis) yields a reflectance map, produced from the data extracted from the EZ Reader for the positive control QUAD1 test strip. Label a show the Area Below Baseline (ABB) for the beta‐lactam drug and label b for the control line. These areas are highlighted in red. Green: the well where the sample is pipetted. Orange: the test lines for quinolones (Q), tetracyclines (T), beta‐lactams (B), and sulfonamides (S). Blue: the control line. The movement of lateral flow is from left to right on both diagrams.

The following method was used for all feather samples. Feathers were first cut into 1 cm pieces and 0.3 g of the sample was weighed into a bijou bottle (Elkay Labs, Basingstoke, UK). A volume of 1.8 mL of negative control buffer (Panel 1 buffer: reference LF‐RFTETQUAD12‐NC10, Panel 2 buffer: reference LF‐QUAD3‐NC10, Charm) was added. The sample was then vortexed for 1 min, centrifuged at 17,000 *g*, and then 300 μL of the supernatant was pipetted into the well of the lateral flow strips (Figure 2) and incubated at 56°C for 5 min before readout.

#### 2.2.1 Processing of the Raw Reflectance Data

Following incubation, test strips were read on an EZ Reader Lite instrument (Charm) and raw data was exported into Excel (Microsoft 2024, v16.82) using the EZReader software (Charm). Reflectance maps were created for each sample for the readout of the relative reflectance (Y‐axis) along the 128 pixels of the strip (X‐axis), referring to the position along the device (Figure 2). The Y‐axis unit, Colour Reflection Unit (CRU), relates to the amount of light the strip reflects at a given pixel, with a white background yielding the highest values as baseline (Figure 2, bottom pane *y*‐axis). The relative difference between the outputs generated by the test lines and the control line on these reflectance maps categorized the sample as “positive” or “negative” for the specific AMD. A CRU greater than the control (i.e., a fainter line) indicates a positive result whereas a CRU lower than the control (i.e., a darker line) indicates a negative result. For each AMD trough abscissa location, the absolute value of the integral of CRU along pixel number was computed as the area below baseline (ABB). Each ABB value was compared to the control line ABB to compute “ΔReflectance” as the difference ABB_control_—ABB_AMD_. The larger the difference between these two values, the higher the concentration of the AMD in the sample, although an exact value (e.g., μg/L) cannot be readily calculated by the instrument. Despite the semi‐quantitative nature of the output, we used ΔReflectance values to determine the limit of detection (LOD) for the analyzed drugs.

### 2.3 Determination of Limits of Detection in Spiked Buffer and Feathers

Drug‐specific dilution series for panel 1 (enrofloxacin, oxytetracycline, ampicillin, sulfadiazine) and panel 2 (streptomycin, neomycin, kanamycin, and spectinomycin) were carried out in spiked negative control buffer for the determination of LOD_*B*_ (μg/L).

#### 2.3.1 Limits of Detection in Buffer

Stock solutions of each AMD were prepared at 5 times the upper bounds of the detection range of LFT in milk (Table 1) and kept at either 4°C or −18°C (Gaugain et al. 2013). The four antimicrobial drugs from each panel were mixed to create one intermediate stock solution matching the upper bounds of the detection range of LFT in milk (Table 1). Intermediate stock solutions were then serially diluted to generate a minimum of ten concentration quartets (Appendixes S2 and S3) and 0.3 μL of each dilution was assayed in triplicate. ∆Reflectance data were analyzed using Prism (version 10.0.0, GraphPad). Non‐linear regressions of ΔReflectance against the logarithms of drug concentrations (μg/L buffer or μg/kg feathers) were performed within the linear response range. The sum of squared differences between observed and predicted values was minimized (Levenberg–Marquardt algorithm) and parameter estimates were determined using profile likelihood analysis. The 95% prediction bands, indicating the area enclosing 95% of data points, was plotted and LOD was estimated as the concentration (μg/L or μg/kg) where the lower bound of the 95% prediction band crossed the *X*‐axis.

**TABLE 1:** Concentration of the stock solution made from analytical standards for panel 1 and 2 (for panel 1, drugs are different from the ones included in the positive control tablet). The drug, drug class, lower and upper bounds of the detection range as described in the Charm documentation and the stock concentration.

Drug name in the intermediate stock solution	Drug class	Concentration of stock solution: 5× upper bounds of the detection range (μg/L)	Lower and upper bounds of the detection range of LFT in milk (μg/L)
Panel 1			
Enrofloxacin	Fluoroquinolone	75	10–15
Oxytetracycline	Tetracycline	350	40–70
Ampicillin	Beta‐Lactam	20	2–4
Sulfadiazine	Sulfonamide	100	10–20
Panel 2			
Streptomycin	Aminoglycoside	875	100–175
Neomycin	Aminoglycoside	1250	175–250
Kanamycin	Aminoglycoside	500	75–100
Spectinomycin	Aminoglycoside	1000	150–200

### 2.4 Determination of Limits of Detection in Spiked Ground Feathers

Similar methods were used for the determination of LOD_*F*_ spiked ground feathers (μg/kg), except for the following matrix‐specific steps.

#### 2.4.1 Procurement of Blank Matrix

Day old specific pathogen‐free (SPF) Lohmann Valo chicks were purchased from the Animal and Plant Health Agency (APHA) and raised for 5 weeks with no exposure to antimicrobials before culling and feather collection (UK Home Office licence PDAAD5C9D). The antibiotic‐free status of these birds was later tested by LFT (as per protocol described) and LC–MS/MS methods (Appendixes S6 and S7).

#### 2.4.2 Blank Feather Spiking and Limit of Detection in Feathers

Frozen blank feather samples (−20°C) were ground using an MPBio Fastprep‐96 (Santa Ana, California, United States) with 30 lysis matrix M beads (MPBio, Santa Ana, California, US), at 1800 oscillations per minute, for a total of 4 min grinding time in 30 s rounds, cooling between rounds on ice. For each drug, 400 μL of the AMD dilutions for panel 1 (Standards A1–J1 Appendix S2) and panel 2 (standards A2–L2 Appendix S3) were spiked onto 0.3 g aliquots of blank ground feathers to make a dilution series. This mixture was then vortex‐mixed for 1 min. Spiked feather powder standards were extracted with 1.4 mL of the relevant negative assay buffer, vortex‐mixed for 1 min, and centrifuged for 1 min (17,000 *g*, Heraeus 3325b Rotor). Each feather extract (0.3 mL) was assayed with LFT in triplicate. The same method was applied to non‐spiked ground blank feather, except that we used 1.8 mL of negative buffer for extraction.

#### 2.4.3 Recovery of AMD Substances

The recovery of AMD substances from feathers was estimated using the ratio of the LOD in buffer ($\mathrm{LO}D_{B}$) to the LOD in feather extracts ($\mathrm{LO}D_{F}$), calculated as follows:

$$\text{Recovery}\;\left(\%\right)=\frac{\mathrm{LO}D_{B}}{\mathrm{LO}D_{F}}\times 100$$

According to the manufacturer's documentation (Charm Sciences), a 1:6 dilution factor is recommended due to the scaling of feather mass to buffer volume. However, as this is an estimated factor, we empirically tested the extraction efficiency in our study by comparing LODs across matrices. While this method does not replace traditional recovery validation through spiked pre‐ and post‐extraction samples, it provides a practical estimate of recoverability within the constraints of field‐applicable lateral flow test (LFT) screening.

### 2.5 Short Term Stability of AMDs

At Day 0, aliquots of buffer were spiked at 2× the estimated LOD_*B*_ and aliquots of blank feathers were spiked at 5× the estimated LOD_*F*_. They were stored either frozen (−20°C) and analyzed on Days 1, 2, 3 (frozen samples stability) or stored at room temperature (~20°C) and analyzed on Day 10 and 30 (room temperature stability). A total of 6 feather samples and 4 buffer samples were analyzed per time point. Differences in ∆Reflectance values between days were analyzed using a non‐parametric Friedman's test, with statistical significance taken as *p* < 0.05.

### 2.6 Cross Validation of a Subset of Samples via Tandem Liquid Chromatography Mass Spectrometry (LC–MS/MS)

#### 2.6.1 Procurement of Feather Samples From Field Studies

A total of 79 feather samples originating from field studies carried out in Bangladesh (ethical approval EC/2020/165/2/1), India (approval URN: 2020 1983‐3) and Vietnam (approval 020‐433/DD‐YTCC) and including 3 negative controls (RVC), were analysed by both LFT and LC–MS/MS methods. Samples were collected from farm and endpoint (market/slaughterhouse) sites from broiler (chickens raised for meat) and layer hens (chickens raised for egg production).

For the first forty samples, freely extractable residues from washed, non‐ground feathers were measured (Wageningen University & Research, Wageningen, Netherlands), using a validated LC–MS/MS panel capable of detecting 48 AMDs (Jansen et al. 2017).

For the second batch of 39 feathers, total residues were measured from ground, unwashed feathers at ANSES (Fougeres, France) using a validated LC–MS/MS panel of 35 AMDs (ANSES, Fougeres, France), based on methods described by Dreano et al. 2021 (where 30 AMDs were included) (Appendix S5). Grinding the feathers ensured the detection of both surface‐bound and keratin‐bound residues, providing insight into the total AMD burden within the feather matrix.

Overall, the two LFT panels encompassed 50 of the AMDs included in LC–MS/MS methods, analyte overlap available in Appendix S5. In cases where trimethoprim (TMP) was detected by LC–MS/MS in the absence of sulfonamide, the sample was classified as sulfonamide‐positive, using TMP as a surrogate for sulfonamide administration. The threshold for an LC–MS/MS positive result was set as > 50 μg/kg.

#### 2.6.2 Statistical Analysis of LC–MS/MS Validation

In this study, we compared the results from the LFT and LC–MS/MS analyses to evaluate the performance of the LFT in detecting AMD residues. LC–MS/MS was used as the gold standard for validation. The comparison aimed to calculate the test sensitivity (Se) and test specificity (Sp) of the LFT, which indicate how well it identifies true positives and true negatives, respectively.

Six antibiotics detected by LC–MS/MS and not reported detectable by LFT (see Charm documentation) were excluded from the comparisons: 2 quinolones (difloxacin, oxolinic acid), 3 sulfonamides (sulfamonomethoxine, sulfamoxole, sulfaphenazole) and Penicillin V. However, drugs detected by LFT and not detectable by LC–MS/MS (due to absence of the specific standard in the LC–MS/SM calibration) could not be edited out from the LFT result (Appendix S5). These instances were treated as false positives (FPs) in the analysis.

We classified test results as follows:

- True Positives (TPs): samples positive by both LFT and LC–MS/MS.
- False Positives (FPs): samples positive by LFT but negative by LC–MS/MS.
- False Negatives (FNs): samples negative by LFT but positive by LC–MS/MS.
- True Negatives (TNs): samples negative by both tests.

To address the performance of the LFT, we calculated the test sensitivity and specificity using the standard formulas:

- Sensitivity (Se) is the probability that a sample with a positive result by LC–MS/MS will also test positive by LFT (Equation 1).
- Specificity (Sp) is the ability of the LFTs to correctly identify negative samples (Equation 2).

(1) $$\mathrm{Se}=\frac{\mathrm{TP}}{\mathrm{TP}+\mathrm{FN}}$$

(2) $$\mathrm{Sp}=\frac{\mathrm{TN}}{\mathrm{TN}+\mathrm{FP}}$$

To estimate the true values of *Se* and *Sp*, we applied Bayesian inference. This allows for the incorporation of prior knowledge and uncertainty in our estimates. We used Beta(1,1) priors, which are uniform distributions, indicating no prior preference for any particular value of sensitivity or specificity. This means that we begin with the assumption that all values for Se and Sp are equally likely, and we update this belief based on the observed data.

The priors are formulated as follows:

$$\mathrm{Se}∼\text{Beta}\left(\mathrm{TP}+1,\mathrm{FN}+1\right)$$

$$\mathrm{Sp}∼\text{Beta}\left(\mathrm{TN}+1,\mathrm{FP}+1\right)$$

This method updates the prior belief with the observed data (TP, FN, TN, FP) and provides posterior distributions for both Se and Sp. The posterior mean estimates represent the most likely value for Se and Sp while the 95% credible intervals (CrIs) provide the range of values within which the true values are likely to lie, with 95% certainty. This interval is computed using the quantile function of the Beta distribution.

$$Se_{\mathrm{CrI}}=q\text{beta}\left(c\left(0.025,0.975\right),\mathrm{TP}+1,\mathrm{FN}+1\right)$$

$$Sp_{\mathrm{CrI}}=q\text{beta}\left(c\left(0.025,0.975\right),\mathrm{TN}+1,\mathrm{FP}+1\right)$$

These estimates were computed for each drug class in the study, where TP, FN, TN, and FP were derived from the lateral flow test (LFT) and the gold standard (LC–MS/MS) data.

## 3 Results

### 3.1 Limit of Detection of Panel 1 and Panel 2 Drugs in Buffer Samples

**FIGURE 3:**
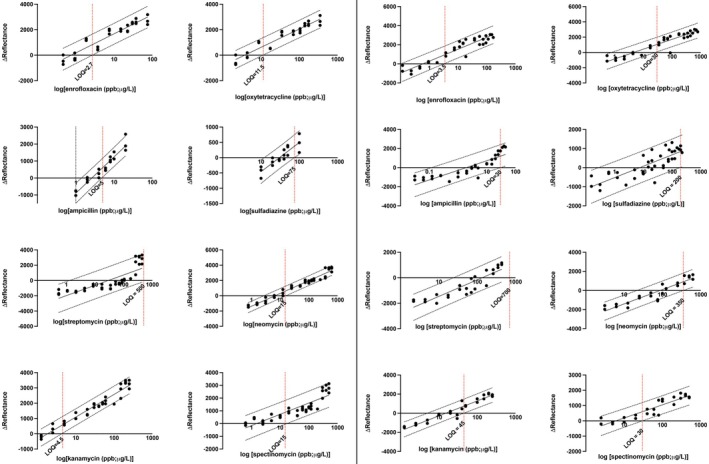
Non‐linear regression model for panel 1 and panel 2 analysis of spiked buffer samples (left panel) and spiked feather samples (right panel). The dilution series results for panel 1 drugs: Enrofloxacin, oxytetracycline, ampicillin, and sulfadiazine and panel 2 drugs: Streptomycin, neomycin, kanamycin, and spectinomycin are shown. Each graph shows the dilution series on a log scale plotted against the difference in the reflection unit of the test line to the control line “∆Reflectance”. Each dilution was conducted in triplicate. The solid black line and the dashed represent the linear regression model and the 95% prediction bands. The limits of detection (LOD; μg/L or μg/kg) for each AMD are shown by a vertical red line and the true value is labelled.

From the 95% prediction bands of the log‐linear regression analysis (Figure 3), the LODs in buffer for the AMDs of panel 1 were 2.7 μg/L for enrofloxacin, 11.5 μg/L for oxytetracycline, 5 μg/L for ampicillin, and 75 μg/L for sulfadiazine. For panel 2 AMD, LODs were 4.5 μg/L for kanamycin, 15 μg/L for neomycin, 15 μg/L for spectinomycin, and estimated around 500 μg/L for streptomycin.

### 3.2 LOD of AMDs From Feather Samples During Extraction

Blank feather samples were negative for panel 1 and panel 2 drugs. For panel 1, LODs in ground feathers were 3.5 μg/kg for enrofloxacin, 30 μg/L for oxytetracycline, 30 μg/kg for ampicillin, and 200 μg/kg for sulfadiazine. For panel 2, LODs were 45 μg/kg for kanamycin, 350 μg/kg for neomycin, 30 μg/kg for spectinomycin, and 700 μg/kg for streptomycin.

### 3.3 Relative Recoveries of Antimicrobial Drug Substances

The estimated recovery of drug substances from feathers was estimated from LOD ratios. Neomycin (3.2%), kanamycin (7.5%), and ampicillin (12.5%) recovered less than the 16.7% estimated from the 1:6 dilution. Sulfadiazine (28.1%) and oxytetracycline (28.8%) all recovered less than a third of the antibiotic on spiked feathers. Spectinomycin recovered just over a third (37.5%) and streptomycin (53.6%) and enrofloxacin (57.9%) recovered more than half of the spiked drugs.

### 3.4 Short Term Stability of AMDs

**FIGURE 4:**
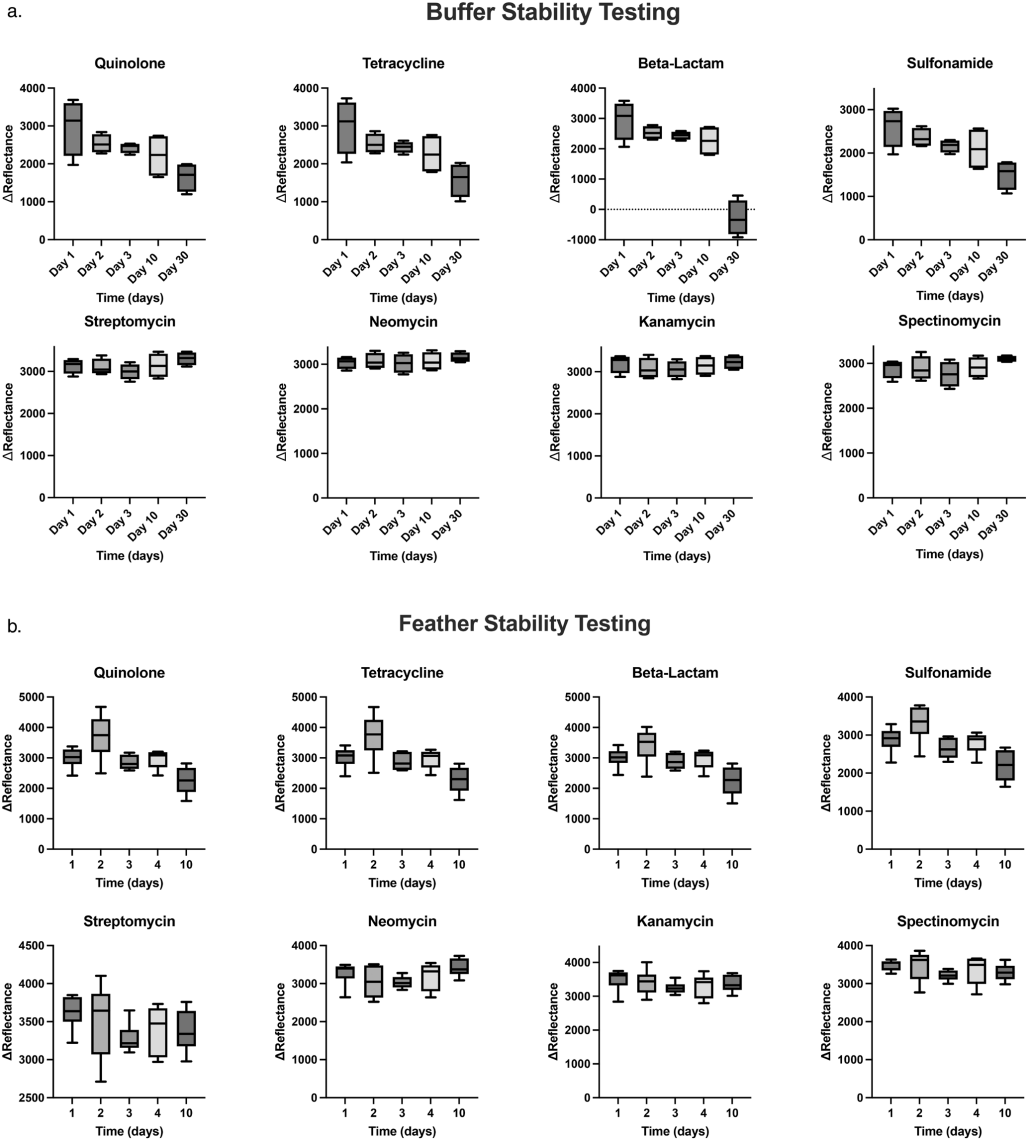
Box plots representing the distribution of the mean and interquartile range (IQR) of the difference in reflectance values (∆Reflectance) between the control and test line for each class/substance of antibiotic for spiked buffer (a) and spiked feathers (b). The lateral flow test panels were assessed on Days 1, 2, 3, 10, and 30 (buffer samples only) after spiking negative control buffer. Samples analyzed on Days 1–3 were maintained under frozen conditions, while samples analyzed on Days 10 and 30 were stored at 20°C (Room Temperature). Only beta‐lactam Day 30 (buffer) showed a significant difference in values (*p* < 0.05).

∆reflectance values from the buffer samples spiked at 2× the estimated LOD_*B*_ showed no significant decrease from Day 0 after 1 freeze/thaw cycle (thawed after 1, 2, and 3 days frozen) and after 10 days at room temperature (Figure 4). On Day 30, beta‐lactams showed a significant decrease in the ∆reflectance value as the samples returned values closer to negative (Friedman tests, *p* < 0.05).

Results from the feather samples spiked at 5× the estimated LOD_*F*_ show little variation over the first 3 days at room temperature. However, at Day 10, there was a reduction in the ∆Reflectance for panel 1 drug classes. Friedman tests on the drug classes in panel 1 and drugs in panel 2 showed no significant (*p* > 0.05) differences between test results over time.

The control line values were also compared over time, and the results of a Friedman test show no significant difference (*p* > 0.05) for each drug class.

### 3.5 Sensitivity and Specificity of LFTs and Predictive Performance

A contingency table showing the number of samples positive/negative for both the LFT and LC–MS/MS panels was created (Table 2). Sensitivity point estimates were between 0.73 and 0.78 for all classes except for beta‐lactams, which were much lower (see Table 3 with 95% CrIs). High specificity was estimated for tetracyclines (0.95) with other classes' estimates ranging between 0.63 and 0.79.

**TABLE 2:** A contingency analysis of the concordance between Lateral Flow Test (LFT) and Liquid Chromatography‐Mass Spectrometry (LC–MS/MS) results for the detection of antimicrobial drugs (AMDs) within four major AMD classes: Quinolones, tetracyclines, beta‐lactams, and sulfonamides. The table displays the counts of samples categorized as LFT‐positive/negative and LC–MS/MS‐positive/negative samples for each AMD class. The LC–MS/MS cut‐off for positive values was 0.1 μg/kg.

	LC–MS/MS positive	LC‐MS/MS negative	Total
Quinolones			
LFT positive	47	7	54
LFT negative	13	12	25
Total	60	19	79
Tetracyclines			
LFT positive	48	1	49
LFT negative	12	18	30
Total	60	19	79
Beta‐Lactams			
LFT positive	2	7	9
LFT negative	11	19	30
Total	13	26	39
Sulfonamides			
LFT positive	33	10	43
LFT negative	12	24	36
Total	45	34	79
Sulfonamides +/or trimethoprim			
LFT positive	37	5	36
LFT negative	13	24	43
Total	50	29	79

**TABLE 3:** The sensitivity, specificity, and 95% credible intervals for the four drug classes: quinolones, tetracyclines, beta‐lactams, and sulfonamides.

Drug class	Se	95% credible interval	Sp	95% credible interval
Quinolones	0.78	0.67–0.86	0.63	0.70–0.90
Tetracyclines	0.78	0.67–0.85	0.95	0.78–0.98
Beta‐Lactams	0.15	0.06–0.34	0.72	0.55–0.84

The ability of LFTs to correctly classify samples relative to LC–MS/MS was highest for tetracyclines, followed by sulfonamides and quinolones, which showed moderate classification performance. In contrast, beta‐lactams exhibited little to no ability to distinguish positive from negative samples, indicating poor reliability for this class.

## 4 Discussion

Here, we present the off‐label use of lateral flow techniques for the detection of antibiotic residues in feather samples. The reliability of this test for their detection is assessed, and test characteristics are defined via cross‐validation with LC–MS/MS. This research contributes to methods of antibiotic characterization in poultry production systems and their use to monitor AMD usage patterns within these systems.

From the results of this study, a feather testing positive for an AMD class would suggest (better than chance) that either the AMD has been administered during the production cycle, or that the chicken has come into contact with the drug through cross‐contamination by other means. This would therefore imply an increased risk of AMR both in the chicken and potentially in the environment.

### 4.1 Storage Stability of AMDs

Results from the stability study indicate that among the eight drugs examined, only beta‐lactams (ampicillin) show significant degradation during mid‐term (30 day) storage at room temperature. The implications of these results suggest that even during a typical broiler production cycle, beta‐lactams may degrade rapidly depending on the time between administration and sampling. This makes beta‐lactam results unreliable in the LFT panel.

For the remaining three drug classes (sulfonamides, tetracyclines, quinolones), there is evidence for at least 30‐day persistence in buffer solutions, suggesting that these drug classes remain stable over time. However, due to limitations in feather availability, stability testing in feather matrices was conducted for a shorter period (10‐days). While previous studies have demonstrated longer persistence of AMDs in feathers post treatment (Chiesa et al. 2018), the current study focused on storage stability persistence, not depletion studies.

It is important to note that while LFTs have demonstrated potential for residue detection in broiler production cycles, their application to longer‐lived poultry such as laying hens requires further investigation. Laying hens have significantly longer lifespans, and AMD exposure patterns differ from those in broilers, particularly in relation to withdrawal periods and residue depletion rates. As an alternative, the adaptation of LFTs for environmental monitoring, such as the detection of AMD residues in poultry house dust, litter, or wastewater, could provide valuable insights into AMD use and exposure in diverse poultry production systems.

### 4.2 Absolute Performance of the Lateral Flow Test (LFT)

For most AMDs, the LOD in buffer was lower than the LOD determined by Charm in milk samples. The LOD of oxytetracycline in buffer (11.5 μg/L) was lower than estimated in previous research (30 μg/L) using similar methods of LFTs (Naik et al. 2017). Previous research on quinolone recovery in feathers using LC–MS/MS methods demonstrated recoveries optimised (with organic solvents) of up to 96.58% for enrofloxacin (Song et al. 2023). Comparable multianalyte LC–MS/MS methods found a higher rate of recovery in enrofloxacin (69%) and sulfadiazine (86%) than we obtained on feather samples (58% and 28% respectively); however, these methods found lower recovery rates for oxytetracycline (5%) and ampicillin (3%) (Dreano et al. 2021). These discrepancies in recovery rates are likely due to the differences in extraction methods (i.e., the type of buffer used and solubility of the drug substance). Overall, using a generic buffer for AMD extraction from feathers allows better yield for all drugs overall than expected from dilution alone, although LC–MS/MS still provides better detection and recovery than the LFTs.

### 4.3 Relative Performance of LFTs Versus LC–MS/MS

Tetracyclines were the only class of AMD with a full match of substances between LFTs and both LC–MS/MS panels (Appendix S5). The reporting limits for Wageningen (first batch) were 10 μg/kg for all tetracyclines, whereas the LODs for ANSES (second batch) ranged between 7 and 15 μg/kg. The LFT panel estimated a LOD for oxytetracycline of 40 μg/kg. The two LC–MS/MS panels differed in one fundamental way; the ANSES method was able to detect both freely extractable and non‐freely extractable drugs (total content), including those embedded within the feather matrix, whereas the Wageningen method only detected freely extractable residues (Jansen et al. 2016). This suggests that, where possible, full extraction during LC–MS/MS is beneficial to explore historical AMD administration; however, for validation of the LFT tests that detect surface AMDs, the freely extractable (i.e., washing) would have been a fairer comparison. Approximately 50% of the total sulfadiazine/trimethoprim and oxytetracycline residues were lost following washing of feathers of broiler chickens housed on the floor, but loss was only 5% for chickens housed in cages (Dréano et al. 2022). This suggests considerable surface contamination of feathers of chickens housed on the floor, thereby underscoring the efficacy of the methodologies described here.

For screening residues of veterinary medicines in the EU, only validated methods with a specificity of at least 95% shall be used for screening purposes (Commission Implementing Regulation (EU) 2021/808 2021). Of the drug classes tested, only tetracyclines would meet these standards. Given the results from our study, it is unlikely that LFT detection methods for quinolones and sulfonamides would be successfully adopted into the governmental sector for residue detection based on results from test specificity.

LFTs offer significant advantages for the monitoring and surveillance of AMD use at the population level, particularly in low‐ and middle‐income countries (LMICs). While LFTs may not be ideal for triaging individual birds as positively or negatively exposed, they provide a practical and cost‐effective method to estimate residue prevalence across large sample sizes, especially in regions with limited LC–MS/MS capacity. Their affordability, ease of deployment, and ability to provide rapid results make them well‐suited for describing AMU patterns across different strata of production systems and for monitoring temporal and spatial changes in AMU practices.

However, while LFTs have these advantages, there are limitations to their accuracy in the absence of fully matched test panels between LFTs and LC–MS/MS. A fully matched test panel would enhance the accuracy of estimates of test Se and test Sp across different AMD classes (excluding tetracycline). The targeted nature of LC–MS/MS, while highly specific and accurate for the drugs included in the panel, inherently limits its scope to detecting only the drugs pre‐specified in the assay. In contrast, the antibody‐epitope recognition mechanism of LFTs could potentially detect unknown or unanticipated drugs of the family. This broader detection capability could theoretically make LFTs more sensitive in identifying residues from drugs not included in the LC–MS/MS assay panel. However, in practice, the relative performance approach used in this study may have penalized the LFT method by misclassifying TPs and FPs when LFT detected AMDs that were not included in the LC–MS/MS panel. Such misclassification could have led to an underestimation of LFT specificity, as highlighted previously (Dubreil et al. 2017).

One limiting factor of the LC–MS/MS cross‐referencing is the lack of aminoglycosides on the LC–MS/MS assays from both laboratories. Previous work validated measurement of aminoglycosides in feathers by LC–MS/MS (Gajda et al. 2019). For streptomycin, recovery rate of 95% with a LOD of 59.4 μg/kg, whereas for spectinomycin recovery was 101% with a LOD of 31.2 μg/kg. The estimated recoveries using the LC–MS/MS methods were much greater than the LFT methods for aminoglycoside extraction and the limit of detection was much lower for streptomycin (933.3 μg/kg) but similar for streptomycin (40 μg/kg). This suggests that with LFT methods, the “rinsing” stage requires refinement to increase the recovery of the drug from the feather matrix, but despite this, the LFTs are likely to be able to detect streptomycin at a lower concentration regardless of the recovery differences.

### 4.4 Applications of LFTs for AMD Residues in Poultry Feathers

Previous research strongly indicates that AMDs persist extensively on poultry feathers. LFTs provide a convenient and effective screening method for monitoring AMD residues, which is crucial for ensuring compliance with agricultural regulatory standards (Groot et al. 2021). However, it is important to clarify that LFTs are not intended for regulatory enforcement or governmental residue monitoring programs. Unlike edible tissues, poultry feathers are not directly consumed and therefore do not require Maximum Residue Limits (MRLs) for food safety compliance. Instead, their value lies in epidemiological assessments, particularly in detecting patterns of AMD exposure that may be linked to AMR in poultry production environments. Such testing is especially valuable in countries where certain AMD classes are banned in agriculture (reserved exclusively for human medicine) or on farms that claim antibiotic‐free status or seek to avoid specific detectable drug classes. Suspect results after screening can be confirmed with LC–MS/MS, but LFTs reduce the initial reliance on external laboratory analysis and enable an immediate characterization of AMD usage at the population level and spatio‐temporal variations among and between production systems.

The use of LFTs for AMD detection in feathers can also be used to establish an epidemiological link between antimicrobial use and exposure in the environment, and antimicrobial resistance (AMR). Feather analysis facilitates the understanding of how AMD usage correlates with residue presence and AMR development in poultry‐associated bacteria (Muaz et al. 2018). This knowledge is crucial for understanding the dynamics of AMR spread and enables the development of evidence‐based strategies to promote responsible AMD use and combat AMR threats.

Building on the inherent advantages of lateral flow tests (LFTs) for rapid on‐site screening, their time efficiency, portability, and cost‐effectiveness make them ideal for routine antibiotic monitoring in agriculture. Unlike LC–MS/MS, which is costly and requires extensive laboratory infrastructure, LFTs offer a practical alternative that allows for more frequent, widespread testing. They enable farmers and regulators to conduct preliminary tests, quickly identifying cases that require more thorough verification via LC–MS/MS. This aligns well with risk‐based monitoring strategies, which focus resources on high‐risk or high‐usage cases, and supports the broader goal of reducing antibiotic usage and combating antibiotic resistance (Groot et al. 2021). Even with lower sensitivity and specificity compared to LC–MS/MS, LFTs serve as an effective, first‐line screening tool that can characterize and describe patterns in antibiotic use within animal agriculture.

## 5 Conclusions

In conclusion, the off‐label use of LFTs demonstrates potential as a rapid method for detecting antimicrobial residues, particularly quinolones, tetracyclines, and sulfonamides, on the surface of poultry feathers. LFTs are ideal for rapid on‐site assessments, providing immediate insights into the presence of antimicrobial residues, which can help guide subsequent, more detailed characterisation using confirmatory methods such as LC–MS/MS. However, it is important to recognise the inherent limitations of LFTs, including the potential for false positives and low specificity. These constraints mean LFTs should not be used as a standalone regulatory tool but rather as a preliminary screening method to identify suspect samples that require further validation. This makes them a valuable component in a multi‐tiered approach to residue detection, enabling efficient resource allocation by pinpointing samples that require comprehensive evaluation.

The validation process highlights the limitations of LFTs, such as their provision of qualitative or semi‐quantitative outcomes. While their reduced specificity may lead to false positives, their ability to detect residues that might be missed by targeted LC–MS/MS panels suggests a complementary role rather than a direct replacement for conventional methods. Within a broader testing network, LFTs can contribute to improved surveillance efficiency by enabling large‐scale screening and prioritization of high‐risk cases for confirmatory testing.

Beyond the poultry sector, similar lateral flow tests could potentially be adapted for other matrices, including animal by‐products, wastewater, and soils, broadening their application for environmental and food safety monitoring. However, any future deployment should carefully consider the trade‐offs between speed, accessibility, and the inherent limitations in accuracy and further work into improved extraction methods.

## Author Contributions

S.H. designed the study, performed the statistical analysis, and drafted the manuscript. S.M. and D.H.‐P. carried out the LC–MS/MS analyses and supported its interpretation. D.P.B. and G.F. supported data interpretation and provided critical revisions to the manuscript. L.P. helped design the study, contributed to data analysis, and contributed to writing the manuscript. All authors contributed to and approved the final version of the manuscript.

## Ethics Statement

The authors declare that all work was carried out in accordance with the applicable ethical guidelines as determined per country of origin; Bangladesh (approval EC/2020/165/2/1), India (approval URN: 2020 1983‐3) and Vietnam (approval 020‐433/DD‐YTCC). All work was reviewed by the Clinical Research Ethical Review Board (CRERB; URN 2020 1983‐2) of the RVC and complied with European standards for the protection of animals. Birds raised at RVC were kept under Home Office licence PDAAD5C9D; their use was approved by the Animal Welfare Ethical Review Body (AWERB) of the RVC.

## Conflicts of Interest

The authors declare no conflicts of interest.

## Supporting information


Appendixes S1–S7.

## Data Availability

The data that support the findings of this study are available from the corresponding author upon reasonable request.
