# Supplementary material for: Validation and Evaluation of Lateral Flow Tests for the Detection of Antimicrobial Residues on Poultry Feathers
Source: J Vet Pharmacol Ther. 2025 Jun 3;48(5):405–16. doi: 10.1111/jvp.70000 (PMC12415806; doi:10.1111/jvp.70000)
Supplement: Supplementary file 1 — Appendixes S1–S7. [file JVP-48-405-s001.docx]

Appendix 1. Composition of experimental drug stock solutions, storage conditions, and expected stability. Data on storage condition stability were obtained from SOP ANSES/LVM/16/02 version 4, June 2019 and Gaugain, Chotard and Verdon (2013).

| **Drug** | **Drug Class** | **Solvent according to GLS** | **Storage Temperature** | **Stability at Storage** |
| --- | --- | --- | --- | --- |
| Enrofloxacin | Quinolone | Water + NaOH | -18 °C | 1 year |
| Oxytetracycline | Tetracycline | Water | -18 °C | 1 year |
| Ampicillin | Beta-Lactam | Water | +4 °C | 1 year |
| Sulfadiazine | Sulfonamide | Water | -18 °C | 6 months |
| Streptomycin | Aminoglycoside | Water | -18 °C | 14 months |
| Neomycin | Aminoglycoside | Water | +4 °C | 14 months |
| Kanamycin | Aminoglycoside | Water | -18 °C | 14 months |
| Spectinomycin | Aminoglycoside | Water | -18 °C | 14 months |

Appendix 2. The dilution series for the panel 1 drugs (enrofloxacin, oxytetracycline, ampicillin, and sulfadiazine) for the experimental validation of the lateral flow test (LFT) strips in spiked buffer (µg/L) and feather (µg/kg) samples.

|  |  | Enrofloxacin | | Oxytetracycline | | Ampicillin | | Sulfadiazine | |
| --- | --- | --- | --- | --- | --- | --- | --- | --- | --- |
| Dilution Relative to Stock | Standard | Buffer (µg/L) | Feather (µg/kg) | Buffer (µg/L) | Feather (µg/kg) | Buffer (µg/L) | Feather (µg/kg) | Buffer (µg/L) | Feather (µg/kg) |
| 2.25 | A1 | 168.8 | 225.0 | 787.5 | 1050.0 | 45.0 | 60.0 | 225.0 | 300.0 |
| 2 | B1 | 150.0 | 200.0 | 700.0 | 933.3 | 40.0 | 53.3 | 200.0 | 266.7 |
| 1.750000 | C1 | 131.3 | 175.0 | 612.5 | 816.7 | 35.0 | 46.7 | 175.0 | 233.3 |
| 1.500000 | D1 | 112.5 | 150.0 | 525.0 | 700.0 | 30.0 | 40.0 | 150.0 | 200.0 |
| 1.250000 | E1 | 93.8 | 125.0 | 437.5 | 583.3 | 25.0 | 33.3 | 125.0 | 166.7 |
| STOCK | F1 | 75.0 | 100.0 | 350.0 | 466.7 | 20.0 | 26.7 | 100.0 | 133.3 |
| 0.500000 | G1 | 37.5 | 50.0 | 175.0 | 233.3 | 10.0 | 13.3 | 50.0 | 66.7 |
| 0.250000 | H1 | 18.8 | 25.0 | 87.5 | 116.7 | 5.0 | 6.7 | 25.0 | 33.3 |
| 0.200000 | I1 | 15.0 | 20.0 | 70.0 | 93.3 | 4.0 | 5.3 | 20.0 | 26.7 |
| 0.150000 | J1 | 11.3 | 15.0 | 52.5 | 70.0 | 3.0 | 4.0 | 15.0 | 20.0 |
| 0.100000 | K1 | 7.5 | 10.0 | 35.0 | 46.7 | 2.0 | 2.7 | 10.0 | 13.3 |
| 0.050000 | L1 | 3.8 | 5.0 | 17.5 | 23.3 | 1.0 | 1.3 | 5.0 | 6.7 |
| 0.025000 | M1 | 1.9 | 2.5 | 8.8 | 11.7 | 0.5 | 0.7 | 2.5 | 3.3 |
| 0.012500 | N1 | 0.9 | 1.3 | 4.4 | 5.8 | 0.3 | 0.3 | 1.3 | 1.7 |
| 0.006250 | O1 | 0.5 | 0.6 | 2.2 | 2.9 | 0.13 | 0.2 | 0.6 | 0.8 |
| 0.003125 | P1 | 0.2 | 0.3 | 1.1 | 1.5 | 0.06 | 0.1 | 0.3 | 0.4 |

Appendix 3. The dilution series for the panel 2 drugs (streptomycin, neomycin, kanamycin, and spectinomycin) for the experimental validation of the lateral flow test (LFT) strips in spiked buffer (µg/L) and feather (µg/kg) samples.

|  |  | Streptomycin | | Neomycin | | Kanamycin | | Spectinomycin | |
| --- | --- | --- | --- | --- | --- | --- | --- | --- | --- |
| Dilution Relative to Stock | Standard | Buffer (µg/L) | Feather (µg/kg) | Buffer (µg/L) | Feather (µg/kg) | Buffer (µg/L) | Feather (µg/kg) | Buffer (µg/L) | Feather (µg/kg) |
| STOCK | A2 | 875.0 | 1166.7 | 1250.0 | 1666.7 | 500.0 | 666.7 | 1000.0 | 1333.3 |
| 0.5 | B2 | 437.5 | 583.3 | 625.0 | 833.3 | 250.0 | 333.3 | 500.0 | 666.7 |
| 0.4 | C2 | 350.0 | 466.7 | 500.0 | 666.7 | 200.0 | 266.7 | 400.0 | 533.3 |
| 0.3 | D2 | 262.5 | 350.0 | 250.0 | 333.3 | 150.0 | 200.0 | 300.0 | 400.0 |
| 0.2 | E2 | 175.0 | 233.3 | 187.5 | 250.0 | 100.0 | 133.3 | 200.0 | 266.7 |
| 0.12 | F2 | 105.0 | 140.0 | 125.0 | 166.7 | 60.0 | 80.0 | 120.0 | 160.0 |
| 0.1 | G2 | 87.5 | 116.7 | 62.5 | 83.3 | 50.0 | 66.7 | 100.0 | 133.3 |
| 0.08 | H2 | 70.0 | 93.3 | 31.3 | 41.7 | 40.0 | 53.3 | 80.0 | 106.7 |
| 0.06 | I2 | 52.5 | 70.0 | 15.6 | 20.8 | 30.0 | 40.0 | 60.0 | 80.0 |
| 0.04 | J2 | 35.0 | 46.7 | 7.8 | 10.4 | 20.0 | 26.7 | 40.0 | 53.3 |
| 0.02 | K2 | 17.5 | 23.3 | 3.9 | 5.2 | 10.0 | 13.3 | 20.0 | 26.7 |
| 0.01 | L2 | 8.8 | 11.7 | 2.0 | 2.6 | 5.0 | 6.7 | 10.0 | 13.3 |
| 0.005 | M2 | 4.4 | 5.8 | 1.6 | 2.1 | 2.5 | 3.3 | 5.0 | 6.7 |
| 0.0025 | N2 | 2.2 | 2.9 | 0.8 | 1.0 | 1.3 | 1.7 | 2.5 | 3.3 |

Appendix 4. The levels of each tetracycline substance present in false negative samples from the Wageningen panel. 0: negative, 1: 0.1-50 (yellow), 2: 50-300 (blue), 3: 300-500 (green), 4: 500-1000 (grey), 5: 1000-5000 (orange), 6: >5000 µg/kg (red).

| **Sample ID** | **LFT (Panel 1)** | **Tetracycline (**LC-MS/MS**)** | **Doxycycline (**LC-MS/MS**)** | **Oxytetra-cycline**  **(**LC-MS/MS**)** | **Chlortetra-cycline**  **(**LC-MS/MS**)** |
| --- | --- | --- | --- | --- | --- |
| VNM-F-01 | 0 | 1 | 1 | 0 | 0 |
| VNM-F-02 | 0 | 1 | 2 | 2 | 0 |
| VNM-F-03 | 0 | 0 | 1 | 0 | 0 |
| VNM-F-04 | 0 | 0 | 1 | 0 | 0 |
| VNM-M-01 | 0 | 1 | 1 | 3 | 0 |
| VNM-M-02 | 0 | 1 | 2 | 2 | 0 |
| IND-F-01 | 0 | 0 | 1 | 2 | 0 |
| BGD-M-01 | 0 | 2 | 1 | 1 | 0 |
| BGD-L-01 | 0 | 2 | 4 | 6 | 0 |
| BGD-L-02 | 0 | 0 | 0 | 5 | 0 |
| BGD-L-03 | 0 | 1 | 1 | 3 | 0 |
| BGD-L-04 | 0 | 1 | 5 | 1 | 0 |

VNM: Vietnam, BGD: Bangladesh, IND: Gujarat, F: Farm, M: Market, L: Layer

Appendix 5. A list of antimicrobial substances included on the Lateral Flow Test (LFT) panel (blue: panel 1, green: panel 2), the LC-MS/MS panel from Wageningen analysis with reporting limit and the LC-MS/MS panel from ANSES analysis with Limit of Detection (LOD) and Limit of Quantification (LOQ), and meat residue LC-MS/MS methods. ‘Y’ indicates inclusion in the specified panel and ‘N’ indicates not detectable by methods.

|  | **Drug Substance** | **Lateral Flow Panel** | **Wageningen Reporting Limit Feathers (µg/kg)** | **ANSES Limit of Detection Feathers (µg/kg)** | **ANSES Limit of Quantification Feathers (µg/kg)** |
| --- | --- | --- | --- | --- | --- |
| **Quinolones** | Ciprofloxacin | Y (QUAD 1) | 10 | 15 | 50-200 |
|  | Danofloxacin | Y (QUAD 1) | 10 | 15 | 50-200 |
|  | Difloxacin | - | 10 | 45 | 150-600 |
|  | Enrofloxacin | Y (QUAD 1) | 10 | 15 | 50-200 |
|  | Flumequin | Y (QUAD 1) | 10 | 28 | 125-500 |
|  | Lomefloxacin | Y (QUAD 1) | - | - | - |
|  | Marbofloxacin | Y (QUAD 1) | 10 | - | - |
|  | Nalidixic Acid | Y (QUAD 1) | 10 | - | - |
|  | Ofloxacin | Y (QUAD 1) |  | - | - |
|  | Oxolinic Acid | - | 10 | 8 | 25-100 |
|  | Norfloxacin | Y (QUAD 1) | 10 | - | - |
|  | Pefloxacin | Y (QUAD 1) | - | - | - |
|  | Orbifloxacin | Y (QUAD 1) | - | - | - |
|  | Sarafloxacin | - | 10 | 15 | 50-200 |
| **Tetracyclines** | Chlortetracycline | Y (QUAD 1) | 10 | 11 | 50-200 |
|  | Epi-Chlortetracycline | - | - | 15 | 50-200 |
|  | Doxycycline | Y (QUAD 1) | 10 | 15 | 50-200 |
|  | Oxytetracycline | Y (QUAD 1) | 10 | 7 | 50-200 |
|  | Epi-Oxytetracycline | - | - | 15 | 50-200 |
|  | Tetracycline | Y (QUAD 1) | 10 | 7 | 50-200 |
|  | Epi-Tetracycline | - | - | 15 | 50-200 |
| **Beta-Lactams** | Amoxicillin | Y (QUAD 1) | - | - | - |
|  | Ampicillin | Y (QUAD 1) | - | - | - |
|  | Cefacetrile | Y (QUAD 1) | - | - | - |
|  | Cefalexin | Y (QUAD 1) | - | - | - |
|  | Cefalonium | Y (QUAD 1) | - | - | - |
|  | Cefazolin | Y (QUAD 1) | - | - | - |
|  | Cefoperazone | Y (QUAD 1) | - | - | - |
|  | Cefquinome | Y (QUAD 1) | - | - | - |
|  | Ceftiofur | Y (QUAD 1) | - | - | - |
|  | Cefuroxime | Y (QUAD 1) | - | - | - |
|  | Cephapirin | Y (QUAD 1) | - | - | - |
|  | Cloxacillin | Y (QUAD 1) | - | 45 | 150-600 |
|  | Dicloxacillin | Y (QUAD 1) | - | 45 | 150-600 |
|  | Oxacillin | Y (QUAD 1) | - | 45 | 150-600 |
|  | Penicillin G | Y (QUAD 1) | - | 4 | 25-100 |
|  | Penicillin V | - | - | 2 | 13-50 |
| **Sulfonamides** | Sulfacetamide | Y (QUAD 1) | 2.5 | - | - |
|  | Sulfachlorpyridazine | Y (QUAD 1) | 2.5 | - | - |
|  | Sulfadiazine | Y (QUAD 1) | 2.5 | 5 | 50-200 |
|  | Sulfadimethoxine | Y (QUAD 1) | 2.5 | 11 | 50-200 |
|  | Sulfadimerazine = Sulfadimidine (Sulfamethazine) | Y (QUAD 1) | 2.5 | 6 | 50-200 |
|  | Sulfadoxine | Y (QUAD 1) | 2.5 | - | - |
|  | Sulfamerazine | Y (QUAD 1) | 2.5 | - | - |
|  | Sulfamethizole | Y (QUAD 1) | 2.5 | - | - |
|  | Sulfamethoxypyridazine | Y (QUAD 1) | 2.5 | 9 | 50-200 |
|  | Sulfamonomethoxine | - | 2.5 | - | - |
|  | Sulfamoxole | - | 2.5 | - | - |
|  | Sulfamethoxazole | Y (QUAD 1) | 2.5 | 14 | 50-200 |
|  | Sulfaphenazole | - | 2.5 | - | - |
|  | Sulfapyridine | Y (QUAD 1) | 2.5 | - | - |
|  | Sulfaquinoxaline | Y (QUAD 1) | 2.5 | 15 | 50-200 |
|  | Sulfathiazole | Y (QUAD 1) | 2.5 | - | - |
|  | Sulfethoxypyridazine | Y (QUAD 1) |  | - | - |
|  | Sulfisoxazole | Y (QUAD 1) | 2.5 | - | - |
| **Diamino-pyrimidine** | **Trimethoprim** | - | 2.5 | 6 | 25-100 |

Appendix 6. Description of the analytical methods for the quantification of antimicrobial drugs in feathers for the 40 field samples submitted to Wageningen Research as conducted by Jansen *et al.* 2017.

For a full description of the methods see Jansen *et al.* (2017). In summary, LC/MS grade antimicrobial standards were purchased from Sigma-Aldrich (St Louis, MO, USA), Toronto Research Chemicals (Toronto, ON, Canada), Council of Europe (EDQM, Strasbourg, France), Santa Cruz Biotechnology (Dallas, TX, USA), Dr. Ehrensorfer GMBH (Augsburg, Germany), ECO Animal Health (London, UK), MSD Animal Health (Boxmeer, The Netherlands), and TCI Europe (Zwijndrecht, Belgium). All drugs were prepared as per manufacturer recommendations, with stock solutions created for various antibiotic classes at specified concentrations. The internal standards were purchased from Witega (Berlin, Germany), Toronto Research Chemicals, Santa Cruz Biotechnology, Sigma Aldrich and MSD Animal Health. A mixture of these standards and internal standards solution were prepared for analysis.

Feather samples were weighed (~1 g) into a 50 mL polypropylene (PP) centrifuge tube (Greiner Bio-One, Alphen aan de Rijn, The Netherlands) and the standards plus 2 mL of 0.125 % TFA in MeOH was added to all samples. The samples were shaken thoroughly by hand and 16mL of McIlvain-EDTA buffer was added. Further shaking for 60 min using a rotary tumbler (Heidolph REAX-2, Schwabach, Germany) took place and samples were centrifuged for 5 min at 3,500 g.

Samples were cleaned by passing through a Strata-X reversed-phase polymeric SPE cartridge (Phenomenex, Torrance, CA, USA) with 5 mL MeOH and subsequently 5 mL water. After rinsing the cartridges with 5 mL of water and applying a dry vacuum for 1 minute, the residues were eluted 5 mL MeOH into a 14 mL glass tube. The solvent was evaporated (40 °C, N_2_) using a TurboVap LV Evaporator (Zymark, Hopkinton, MA, USA) and the residues were reconstituted in 200μL MeOH by using a vortex mixer (IKA, Staufen, Germany). They were further diluted with 300 μL water and transfered into a glass vial suitable for LC-MS/MS analysis.

The UHPLC system (Waters, Milford, MA, USA) features an Acquity HSS-T3 C18 analytical column (2.1 × 100 mm, 1.7 μm) at 30 °C. The mobile phase consists of 2 mM ammonium formate and 0.16 % formic acid in water (Solvent A) and MeOH (Solvent B). Gradient: 0–1.0 min, 0 % B; 1.0–2.5 min, 25 % B; 2.5–5.4 min, 70 % B; 5.4–5.5 min, 100 % B, hold 1.0 min. Flow rate: 0.4 mL min−1; injection volume: 5 μL. Detection: MS/MS (Xevo TQS, Waters) in ESI mode. Operating parameters: capillary voltage 3.0 kV, source temperature 130 °C, desolvation temperature 450 °C, cone gas flow 150 L h−1, desolvation gas flow 650 L h−1. Data processed using MassLynx 4.1 software.

Appendix 7. Description of the analytical methods for the quantification of antimicrobial drugs in feathers for the 39 field samples submitted to ANSES as conducted by Dréano *et al.* 2021.

The full detailed analytical procedure of the multi-class LC-MS method has been described in Dréano *et al.* 2021. Briefly, 0.5 g of each finely ground feather sample spiked with 200 μL of a mixed solution of isotopically labelled internal standards were extracted by a solid-liquid procedure and salting-out via consecutive addition to the samples of Na2EDTA, 2 mL purified water, 4 mL acetonitrile, 30 μL formic acid and the mixture of Na2SO4, NaCl and dehydrated trisodium citrate. After stirring on a rotary shaker for 10 min, supernatants were collected and centrifuged at 3000 g for 10 min at 4 °C, then evaporated under nitrogen at 50 °C with DMSO. Extracts were reconstituted in 900 μL of 0.2 M ammonium acetate. After the addition of 1 mL isooctane, the lower phase was filtered to 0.22 μm after centrifugation at 20,000 g for 5 min at 4 °C and transferred to an LC vial prior to analysis.

Analytes were detected and quantified in the extracts by LC-ESI-MS/MS (Shimadzu LC-20AD-XR system (Kyoto, Japan), Sciex API5500 triple quadrupole MS (San Jose, USA)). A Kinetex C18 column (100 × 2.1 mm, 2.6 μm) protected with a C18 security guard column (4 × 2 mm) from Phenomenex (California, USA) were used for the separation of the analytes. Purified water (A) and acetonitrile (B), both acidified with 0.2 % of formic acid were used as mobile phases. Electrospray ionization in positive mode was performed.

MS detection was carried out in MRM scheduled mode. Analyst v.1.7.3 and Multiquant v.3.0.3 were used for system control and data processing, respectively. The concentrations of antimicrobial agents were calculated by linear or quadratic regression using calibration curve performed with matrix-fortified calibration standards (CS) covering the validated dosing range of each respective analyte. Quality control (QC), i.e., blank matrix and procedural blanks were prepared and analysed during each sequence runs to check the absence of background contamination.
